# Connective Tissue Growth Factor in Regulation of RhoA Mediated Cytoskeletal Tension Associated Osteogenesis of Mouse Adipose-Derived Stromal Cells

**DOI:** 10.1371/journal.pone.0011279

**Published:** 2010-06-23

**Authors:** Yue Xu, Diane R. Wagner, Elena Bekerman, Michael Chiou, Aaron W. James, Dennis Carter, Michael T. Longaker

**Affiliations:** 1 Hagey Pediatric Regenerative Medicine Laboratory, Department of Surgery, Stanford University School of Medicine, Stanford, California, United States of America; 2 Biomechanical Engineering Division, Department of Mechanical Engineering, Stanford University, Stanford, California, United States of America; 3 Bioengineering Graduate Program and Aerospace and Mechanical Engineering, University of Notre Dame, Notre Dame, Indiana, United States of America; University of Birmingham, United Kingdom

## Abstract

**Background:**

Cytoskeletal tension is an intracellular mechanism through which cells convert a mechanical signal into a biochemical response, including production of cytokines and activation of various signaling pathways.

**Methods/Principal Findings:**

Adipose-derived stromal cells (ASCs) were allowed to spread into large cells by seeding them at a low-density (1,250 cells/cm^2^), which was observed to induce osteogenesis. Conversely, ASCs seeded at a high-density (25,000 cells/cm^2^) featured small cells that promoted adipogenesis. RhoA and actin filaments were altered by changes in cell size. Blocking actin polymerization by Cytochalasin D influenced cytoskeletal tension and differentiation of ASCs. To understand the potential regulatory mechanisms leading to actin cytoskeletal tension, cDNA microarray was performed on large and small ASCs. Connective tissue growth factor (CTGF) was identified as a major regulator of osteogenesis associated with RhoA mediated cytoskeletal tension. Subsequently, knock-down of CTGF by siRNA in ASCs inhibited this osteogenesis.

**Conclusions/Significance:**

We conclude that CTGF is important in the regulation of cytoskeletal tension mediated ASC osteogenic differentiation.

## Introduction

Connective tissue growth factor (CTGF, CCN2), a member of the CCN family of proteins, is a cysteine-rich pro-adhesive matricellular protein that plays an essential role in the formation of blood vessels, bone, and connective tissue [1]. The angiogenic inducer, 61 protein (Cyr61) and connective tissue growth factor (CTGF) are structurally associated with secreted matrix cellular proteins, and function in cell adhesion, migration, proliferation and extracellular matrix (ECM) synthesis [2]. As an example of context-dependent activity, functions of CTGF largely depend on the interactions with other molecules in the microenvironment [3]. Thus, instead of being a growth factor in the original sense of the word, CTGF is better described as a modulator of complex activities for other growth factors. In addition, CTGF induces chondrogenesis and determines osteoblast differentiation [4], [5]. Previous studies have shown that CTGF is highly expressed by osteoblasts, and CTGF null mice exhibit impaired chondrocytic cell proliferation and angiogenesis resulting in neonatal skeletal defects [6]–[9]. These observations suggest that CTGF is important in bone and cartilage physiological events and repair.

Studies reveal that tension in the actin cytoskeleton, which is modulated by the RhoA/Rock signaling pathway, is a key player in many cellular processes including proliferation, differentiation, stabilization of cell-matrix adhesion and modulation of gene expression. For example, diverse events such as branching morphogenesis during lung development [10], [11], and corneal epithelial wound healing are both regulated by cytoskeletal tension through the RhoA/Rock pathway [12]. In another recent study, inhibition of the RhoA/Rock pathway in mesenchymal limb bud cells altered chondrogenic gene expression, indicating that cytoskeletal tension and chondrogenic differentiation are interrelated [13]. One mechanism by which cells regulate the mechanical loads generated by their actin cytoskeleton is through cell morphology; larger cells that are more spread contracted a flexible substrate beneath them, while smaller cells did not. In bone marrow-derived mesenchymal cells (BMSCs), cell shape and cytoskeletal mechanics, mediated through the RhoA/Rock signaling pathway, drove commitment to the osteogenic or adipogenic lineages: large spread cells underwent osteogenesis with high levels of active RhoA, while small unspread cells underwent adipogenisis with limited RhoA activation [14], [15]. Thus, cell size and its associated mechano-environment are key attributes in mesenchymal cell differentiation; however, the precise cellular signaling events that lead to the transition in lineage commitment remain unaddressed.

Mesenchymal cells obtained from adipose tissue contain a large number of progenitor cells with capabilities of osteo-, chondro-, and adipogenic differentiation [16]. Moreover, an inverse relationship between osteogenic and adipogenic commitment within the whole adipose-derived stromal cell (ASC) progenitor pool has been observed [17], [18]. Studies using ASCs offer great promise for skeletal tissue reconstitution and replacement [19], [20]. Thus, understanding the mechanisms involved in the cellular signaling of lineage commitment is an important step toward the regulation of mesenchymal cell differentiation. In this study, we manipulated the *in vitro* cell seeding densities of ASCs, resulting in large and small cells with distinguished microenvironments associated with actin cytoskeletal tension, and subsequently explored the influence on osteogenic and adipogenic differentiation of ASCs. Through the results of a gene array and siRNA knock-down experiments, we determined that CTGF is highly induced in large ASCs and is a pro-osteogenic effector that plays an important role in RhoA mediated cytoskeletal tension-associated osteogenesis.

## Methods

### Chemicals and Medium

Dulbecco's Modified Eagles Medium (DMEM) and penicillin/streptomycin were purchased from Invitrogen, Inc. (Carlsbad, CA). Fetal bovine serum (FBS) was purchased from Omega Scientific, Inc. (Tarzana, CA). All cell culture wares were purchased from Corning Inc, (San Mateo, CA). Unless otherwise specified all other chemicals were purchased from Sigma-Aldrich, (St. Louis, MO). Recombinant CTGF was from ProSpec Protein Specialists (Rehovot, Isreal).

### ASC Harvesting and Seeding

All experiments were performed in accordance with Stanford University Animal Care and Use Committee (IACUC) guidelines. The IACUC protocol number for our study is 9999/7373. Mouse adipose-derived stromal cells (ASCs) were isolated as described previously [16]. ASCs were expanded in growth media containing DMEM (Mediatech, Herndon, VA), 10% FBS (Invitrogen, Carlsbad, CA), 1% penicillin/streptomycin. Growth media were changed every two days and cells were subcultured by trypsin/EDTA. Passage one cells were used for the following experiments. ASCs were seeded in 12-well dishes with different seeding densities; a low density of 1,250 cells/cm^2^; a medium density of 2,500 cells/cm^2^, or a high density of 25,000 cells/cm^2^. Crystal violet staining was performed to show the microscopic cell morphology and measure the cell sizes at different seeding densities. Image J software was utilized to trace the cell shape and calculate the cell size at different density seeding.

### *In Vitro* proliferation

ASCs were seeded at low, medium and high densities as described above. After attachment, cells were cultured in bipotent medium containing both osteogenic and adipogenic factors. At days 1, 3 and 7, cell proliferation was assessed via MTT (3-[4,5-Dimethylthiazol-2-yl]-2,5-diphenyltetrazolium bromide) assay (n = 3 wells per condition). Into each well, 50 µl of 5 mg/ml MTT solution was added and allowed to incubate for three hours at 37°C. The colored formazan product was dissolved in DMSO and absorbance was measured at 570 nm.

### *In Vitro* Osteogenic Differentiation

ASC osteogenesis was induced by treatment with basic osteogenic differentiation medium (ODM) containing DMEM, 10% FBS, 100 µg/ml ascorbic acid, 10 mM β-glycerophosphate, 1% penicillin/streptomycin. The ODM was replenished every three days. After one week of differentiation, early alkaline phosphatase activity staining was performed and quantification of alkaline phosphatase activity was assessed by normalizing to the total protein quantity. After two weeks of osteogenic differentiation, terminal osteogenic differentiation was evaluated by staining the extracellular matrix (ECM) mineralization with Alizarin red S. To inhibit cytoskeletal tension, cytochalasin D (0.5 ug/ml) and Y-27632 (10 uM) were used to the differentiation medium block the actin plymorlization and Rock/Rho pathways, respectively. Recombinant CTGF (100 ng/ml) was supplemented to the differentiation media in order to rescue the cytoskeletal tension.

To assess the effect of secreted factors from different density-seeded cells, condition medium collected from low-, mediun- and high-density-seeded ASCs was used. After attachment (approximately 12 hours post-seeding), media from each seeding density was collected; 100 µg/ml ascorbic acid and 10 mM β-glycerophosphate was added to collected media. In a separate 12-well-dish, in order to observe differences in differentiation with the use of conditioned media, ASCs were seeded at a relatively higher density of 20,000 cells/cm^2^. Upon attachment, the same density-seeded cells were treated with conditioned ODM generated by different density seeding. After one week, early alkaline phosphatase activity staining and quantification were assessed to study the effects of secret factors.

### Staining and Quantification of Alkaline Phosphatase Activity

After one week of osteogenic differentiation, medium was removed and differentiated cells were washed twice with phosphate buffered saline. Cells were then fixed with 60% acetone and 40% Citrate working solution for 30 seconds at room temperature. Following a brief rinse with deionized water, cells were stained with a diazonium salt solution comprised of Fast Violet B (0.024%) and 4% Naphthol AS-MX Phosphate Alkaline Solution (Sigma Aldrich, St. Louis, MO) in the dark for 30 minutes at room temperature. Positively stained cells were observed using phase-contrast microscopy (Leica, San Jose, CA).

Alkaline phosphatase activity of differentiated cells was also determined using a biochemical colorimetric assay kit (Sigma Aldrich, St. Louis, MO) as described by the manufacturer. Briefly, cells were washed with cold phosphate buffered saline. Cells were scraped into a radioimmunoprecipitation assay (RIPA) buffer (containing 50 mM Tris-HCl pH 7.5, 150 mM NaCl, 5% Glycerol, 1 mM EDTA, 1% NP-40, 0.1% SDS and 0.25% Na-deoxycholate) and centrifuged. The enzymatic alkaline phosphatase activity in the supernatant of cell lysate was assayed by measuring the *p*-nitrophenol formed from the enzymatic hydrolysis of *p*-nitrophenylphosphate, used as the substrate, at 405 nm. In order to consider the protein turnover during the differentiation, the quantity of alkaline phosphatase activity was normalized to total protein, as measured by BCA protein assay reagent kit (Pierce, Rockford, IL.). Experiments were performed in triplicate wells and means and standard deviations were calculated. A Student's *t*-test was used to assess significance (*p≤0.05).

### Alizarin Red Staining and Quantification

After two weeks osteogenic differentiation, matrix mineralization and calcium deposition were stained by Alizarin red. Cultured cells were washed briefly with phosphate buffered saline. Next, cells were washed briefly with deionized water, to minimize potential for binding of Alizarin stain to PBS. As well, untreated cells were washed with PBS in the same manner, so as to serve as a control. Next, cells were fixed for 15 minutes in 100% ethanol and stained with 0.2% Alizarin red S solution (PH 6.4) for 30 minutes. Stained cells were extensively washed with deionized water to remove the nonspecific precipitation. The positive red staining represents calcium deposits of matrix formation on the mineralized cells. The matrix mineralization was quantified by extraction of Alizarin red S staining with 100 mM cetylpyridinium chloride solution and measuring the absorbance at 570 nm. Experiments were performed in triplicate wells. Photographs were obtained and presented for the analysis of late stage osteogenic differentiation.

### *In vitro* Adipogenesis and Assessment

ASCs were seeded at different densities as described above. After attachment, adipogenic differentiation was induced with adipogenic differentiation media (ADM) containing 10 µg/ml insulin, 1 µM dexamethasone, 0.5 mM methylxanthine, and 200 µM indomethacin. ADM was replenished with growth media containing 10 ug/ml insulin after three days of differentiation. Adipogenic differentiation was assessed by staining with Oil Red O at one week of differentiation. Briefly, cells were fixed in 10% formalin/PBS for 30 minutes at room temperature and then incubated in 60% Oil Red O solution (0.3% Oil Red O in isopropanol) for 30 minutes in 37°C. Cells that developed lipid accumulation were stained red. Images of adipogenic differentiation were obtained microscopically. The quantification of Oil Red O was performed by extracting the stain with isopropanol and measuring the absorbance at 510 nm. Experiments were performed in triplicate wells. A Student *t*-test was calculated to assess the significance (*p≤0.05).

The bipotent media, which was used to provide an environment for both lineage differentiation, contained both osteo- and adipogenic components [16].

### Micropatterning: Stretching Cells in Defined Cell Sizes

In order to verify the expression of CTGF affected by cell size, we applied micropatterning and managed seeding cells with defined sizes [21]. Briefly, polydimethylsiloxane (PDMS) stamps with different size of islands (ranging from 10 um∼100 um in diameter) were immersed in fibronectin at a concentration of 50 µg/ml in PBS for one hour, and allowed to dry. The stamps were then placed in contact with the non-coated dish surface for at least five seconds before being peeled off. The entire surface was subsequently immersed in pluronic F-127 (0.2% w/v) in PBS for three hours at room temperature in order to block the nonprinting area [22]. Following a brief rinse with deionized water, ASCs were seeded on the surface and allowed to settle on the fibronectin (final concentration of 100 ug/ml in PBS) printed area. After crystal violet staining, ASCs were observed to be spread at sizes that corresponded to the printed areas. Only single cells landed in the defined printed area were analyzed by immunofluorescence.

### Immunofluorescence

Cells were fixed with 4% paraformaldehyde/4% sucrose and were blocked with a non-protein blocker (Dakocytomation, Carpinteria, CA) for one hour at 37°C, and incubated with primary antibodies (CTGF and RhoA were from Santa Cruz Biotechnology, Santa Cruz, CA) for overnight at 37°C. Then, a FITC- conjugated secondary antibody (Molecular Probes, Eugene, OR) was applied to the cells for one hour at 37°C. Cells were then mounted using Vectashield fluorescent mounting solution with DAPI (Vector Labs, Burlinghame, CA) and analyzed by fluorescence microscopy at 40× magnification (Carl Zeiss, Thornwood, NY).

### ASC morphology with RhoA/Rock inhibitors

ASCs were seeded at low or high density and treated with cytochalasin D or Y-27632 as above. After 24 hours of treatment, cells were fixed in a 4% paraformaldehyde/4% sucrose solution. Phalloidin conjugated to rhodamine (Molecular Probes, Eugene OR) was applied for one hour at 37°C to visualize F-actin. Cells were then mounted using Vectashield fluorescent mounting solution with DAPI (Vector Labs, Burlingame, CA) and visualized by fluorescence microscopy at 40× magnification (Carl Zeiss, Thornwood, NY).

### RNA isolation and microarray hybridization

Total RNA was isolated using TRIzol solution (Invitrogen™) according to manufacturer's instructions. RNA from three separate treatments of low and high seeding in growth media, and low and high seeding in bipotent media were harvested for microarray analysis. Fluorescently labeled DNA probes were prepared from 50 to 70°C total RNA isolated from low density-seeded and high density-seeded cells (Cy5-labeled) and Universal Human Reference RNA (Stratagene, La Jolla, CA) (Cy3-labeled) by reverse transcription using an Oligo dT primer 50-TTTTTTTTTTTTTTT-30 (Qiagen, Valencia, CA) as described [23]. Labeled probes from low and high density seeded cell RNA and reference RNA were mixed and hybridized overnight at 65°C to spotted cDNA microarrays with 41,126 elements (Stanford Functional Genomics Facility, Stanford, CA). Microarray slides were then washed to remove unbound probe and scanned with a GenePix 4000B scanner (Axon Instruments, Inc., Union City, CA).

### Data processing and analysis

The acquired fluorescence intensities for each fluoroprobe were analyzed with GenePix Pro 5.0 software (Axon Instruments, Inc.). Spots of poor quality were removed from further analysis by visual inspection. Data files containing fluorescence ratios were entered into the Stanford Microarray Database (SMD) where biological data were associated with fluorescence ratios and genes were selected for further analysis [24]. Hierarchical clustering was performed by first retrieving only spots with a signal intensity >150% above background in either Cy5- or Cy3 channels in at least 70% of the microarray experiments from SMD. We selected clones whose expression levels varied at least threefold in all three samples. The genes and arrays in the resulting data tables were ordered by their patterns of gene expression using hierarchical clustering analysis, and visualized using Treeview software (http://rana.lbl.gov/EisenSoftware.htm) [25]. Genes with potentially significant differential expression in ASCs from low- and high-density seeding were identified using the Significance Analysis of Microarrays (SAM) procedure, which computes a two-sample T-statistic (e.g., for low-density-seeded cells vs. high-density-seeded cells) for the normalized log ratios of gene expression levels for each gene. We used a selection threshold that gives a relatively low false discovery rate and identifies a relatively high number of significant genes [26]. Array data is publicly accessible at http://www.ncbi.nlm.nih.gov/geo/info/linking.html, accession number GSE19924. All raw data has been deposited in GEO data base.

### Quantitative Real-time Polymerase Chain Reaction

Total RNA was harvested from ASCs of low-density and high-density seeding by using an RNAeasy Mini kit (Qiagen, Valencia, CA), and treated with DNAse I (Ambion, Austin, TX). Reverse transcription was performed using the Taqman^®^ Reverse Transcription Kit from Applied Biosystems, Foster City, CA. Quantitative real-time PCR was carried out using the Applied Biosystems Prism 7900HT Sequence Detection System. The Sequence for CTGF gene (NM_010217) primers is as following, forward, 5-GGGCCTCTTCTGCGATTTC-3; reverse, 5-ATCCAGGCAAGTGCATTGGTA-3. Primers were first tested to determine optimal concentrations, and products were run on a 2% agarose gel to confirm the appropriate size and RNA integrity. Gene expression values were normalized to 18S ribosomal RNA quantity. All reactions were performed in triplicate. Representative graphs are shown with error bars indicating standard deviation of the triplicate reactions. Multiple independent experiments were conducted with similar trends. Statistical analysis was performed using Student's *t* test with *p≤0.05 considered significant.

### siRNA transfection

CTGF knock down experiments were performed by transfection of CTGF siRNA in ASCs, three pairs of the double strand RNA were purchased from Ambion (Ambion, Austin, TX.). According to the real time PCR results, the sequences of 5 -GGUGAUAAAGCUAUGUAUUtt-3, 5-AAUACAUAGCUUUAUCACCtg-3 showed maximal knock-down efficiency of CTGF in cells. ASCs seeded in low- and high-density were transfected with CTGF siRNA (siCTGF), non-silencing siRNA (as a control) (Invitrogen, Carlsbab, CA) or lipofectamine alone (LIPO) as indicated above. Four hours of post-transfection, cells were washed briefly with PBS and ODM was then changed to the transfected cells. Meanwhile, RNA and protein were harvested from the cells of 24 and 48 hours post-transfection for the evaluation of CTGF knock-down. Transfection efficiency was determined by quantitatively assessing gene expression and protein expression of CTGF. At 48 hours post-transfection, 80% of knock-down efficiency was achieved shown by the expression of gene and protein. Transfection was performed in multiple individual wells (N = 6). Experiments were repeated at least three times with different isolation of the cells.

### Statistical analysis

Means and standard deviations were calculated from numerical data. In figures, bar graphs represent means and standard deviation. Student *t*-test was performed to calculate the significance. *P≤0.01 was considered to be significant.

## Results

### Different density seeding results in different sizes of ASCs and influences osteogenesis and adipogenesis

To correlate osteogenesis and adipogenesis to ASC size, cells were seeded in low, medium and high densities and induced to osteogenic and adipogenic differentiation in either osteogenic differentiation media (ODM) or adipogenic differentiation media (ADM), respectively. Bipotent media, which contains both osteogenic and adipogenic components, was used to concurrently observe the bipotent potential of ASCs' differentiation with different seeding density [16]. ASC adipogenesis was assessed by Oil Red O after one week of differentiation. Osteogenesis was assessed at one week based on alkaline phosphatase activity staining and quantification.

**Figure 1:**
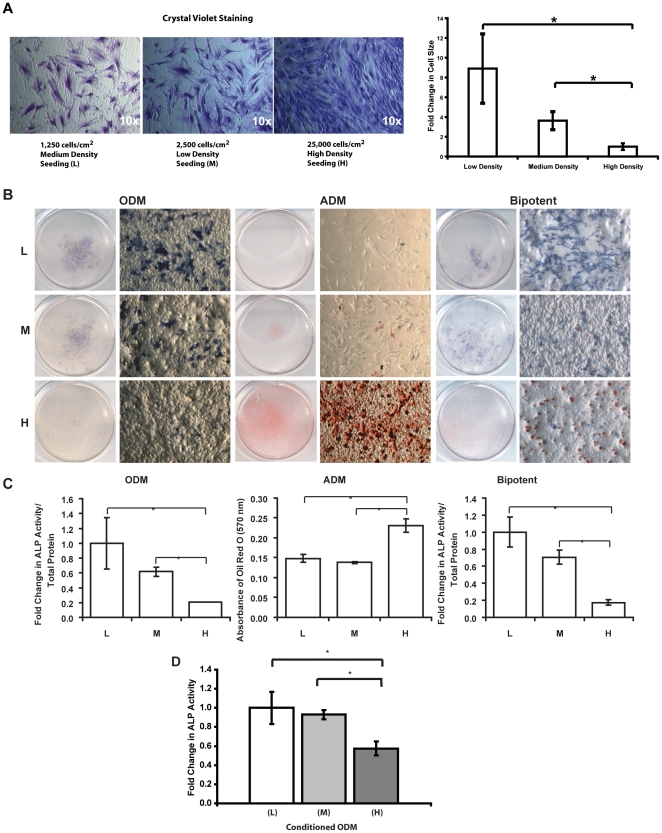
ASC seeding, morphology, cell size, osteogenic and adipogenic differentiation. A) Crystal violet staining of ASCs seeded at different densities. Cells featured large morphology at a low density (L) seeding (1,250 cells/cm^2^) and a medium density (M) seeding (2,500 cells/cm^2^); in contrast, ASCs were compressed into small cells at a high density (H) seeding (25,000 cells/cm^2^). Calculated average cell sizes (n = 10) by Image J software are shown in the graph. The arbitrary number read by Image J for average area of low density seeding was 41752.4, medium density seeding was 26565.7 and high density seeding is 5471.5. Graph shows the fold changes in cell size for low- and medium- density seeded ASCs as compared to high-density seeded cells (*p≤0.05). B) Scanned and microscopic images of cells plated at a low density (L) of 1,250 cells/cm^2^, a medium density (M) of 2,500 cells/cm^2^, or a high density (H) of 25,000 cells/cm^2^ cultured for one week in osteogenic differentiation media (ODM), adipogenic differentiation media (ADM), or a bipotent media containing both osteogenic and adipogenic factors (bipotent). Cells in osteogenic media were stained for alkaline phosphatase activity, cells in adipogenic media were stained with Oil Red O, and cells in bipotent media were stained for both. Substantially higher alkaline phosphatase activity staining was shown in low density and medium density (large cells) seeding conditions in both ODM and bipotent media. Conversely, high-density-seeded small cells committed to adipogenesis in both ADM and bipotent media. C) Quantitative analysis of alkaline phosphatase activity (normalized to total protein content per well) for cells plated at low, medium and high density in ODM and bipotent media and quantification of Oil Red O staining after one week in ADM. Low- and medium-density-seeded ASCs showed significantly higher osteogenic differentiation potential in both ODM and bipotent media as compared to high-density-seeded ASCs after normalizing to the total protein content (n = 3 and *p≤0.05). The extracted Oil Red O from high-density-seeded cells was significantly higher than the low- and medium-density-seeded cells (n = 3 and *p≤0.05). D) Paracrine regulatory effect of osteogenesis by different density seeded cells. Although ASCs were seeded at same density of 20,000 cells/well, alkaline phosphatase activity staining and quantification showed abundant staining in ASCs treated with condition medium generated by low- and medium- density seeded cells as compared to high density-seeded cells (n = 3 and *p≤0.05).

As demonstrated by crystal violet staining, ASCs spread into large cells when they were seeded at a lower density (1,250 cells/cm^2^) and a medium density (2,500 cells/cm^2^); conversely, when ASCs were seeded at high density (25,000 cells/cm^2^), they were smaller in size microscopically (**Figure 1A**). To quantitatively determine the relationship between the cell size and seeding density, we utilized Image J software, and traced 10 different cells from the pictures taken from low-, medium- and high- density seeding. Average areas of the cells from different seeding density were calculated separately. Cells seeded at low- and medium- density were significantly larger in size as compared to the cells seeded at high-density (*p≤0.05).

Upon osteogenic induction, enhanced alkaline phosphatase activity staining was observed in the low- and medium-density-seeded, larger cells at one week of early osteogenesis (**Figure 1B**). A quantitative alkaline phosphatase activity assay was performed in order to consider the cell number presented in different density-seeded samples. Data demonstrated significantly higher alkaline phosphatase activity in the low- and medium-density-seeded cells as compared to the high-density-seeded cells cultured in osteogenic medium for one week (*p≤0.05) (**Figure 1C**). Conversely, robust adipogenesis was observed by Oil Red O staining in the high-density-seeded, smaller cells after one week of adipogenic differentiation (**Figure 1B**). The quantification of Oil Red O demonstrated significant differences between the high- density-seeded and low-/medium- density-seeded cells (*p≤0.05) (**Figure 1C**). Thus, enhanced osteogenesis was observed in larger cells versus significant adipogenesis in the high-density-seeded, smaller cells (*p≤0.05). We found that Alizarin Red staining of late stage mineralization correlated with the alkaline phosphatase activity staining (data not shown). Similarly, when ASCs were cultured in bipotent differentiation medium with different density seeding, cell size affected osteogenesis and adipogenesis with the same trend. Therefore, we observed that cell size was tightly associated with the outcome of mesenchymal cell differentiation.

Increasing evidence suggests that mechanical deformation due to shear forces or cell spreading plays an important role in differentiation by influencing cell function. The hypothesis is that growth factors and cytokines induced by such mechanical strain act as modulators stimulating cell differentiation within the microenvironment. To confirm the paracrine regulatory mechanism, ASCs were seeded at the same density of 20,000 cells/cm^2^. Subsequently, conditioned media generated by low-, medium- and high-density-seeded cells were used to induce osteogenesis in these ASCs seeded at the same density. After differentiation, alkaline phosphatase activity staining and quantification showed that large, spreading cells secreted growth factors and cytokines in the conditioned medium that contribute to osteogenic differentiation *in vitro* when ASCs were seeded at the same density (**Figure 1D**). These results demonstrated this mechanical stimulus regulated mesenchymal cell differentiation via a paracrine mechanism.

### Expression of Connective Tissue Growth Factor (CTGF) Associated with Cell Size

**Figure 2:**
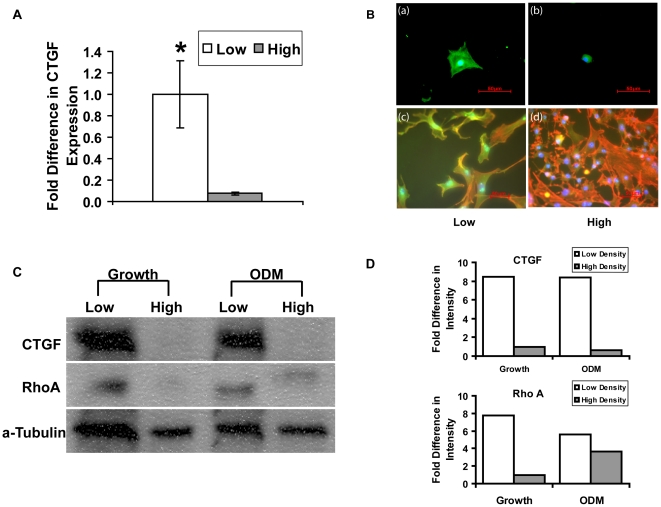
Increased CTGF expression in low-density-seeded, large cells. A) Quantitative real-time PCR demonstrated significantly higher levels of CTGF expression in low-density-seeded cells as compared to high-density-seeded cells (*p≤0.05). Values were normalized to the expression in high-density-seeded cells. B) Immunofluorescent images of CTGF staining. ASCs spread in a large microprinted (larger than 50 um in diameter) area (a) showed intense staining of CTGF (green) as compared to ASCs spread in a small printed (smaller than 20 um in diameter) area (b). DAPI (blue) counter staining indicates nuclei. RhoA and cytoskeletal filaments stained by rhodamine-conjugated phalloidin (staining for F-actin) at low (c) or high (d) density. Cells were simultaneously stained for F-actin (red) and for RhoA with a FITC-conjugaed anti-RhoA (green) antibody and counterstained with DAPI for cell nuclei (blue). ASCs seeded at low density displayed a higher expression of RhoA whereas ASCs seeded at high density showed more overt actin ruffles within each cell. C) Western blot confirmed the changes in protein expression for both CTGF and RhoA. Abundant expression of CTGF was shown in low-density-seeded ASCs after attachment in growth media (Growth); the difference remained significant when osteogenesis was induced by ODM. In addition, the expression of cytoskeletal protein, RhoA was also higher in low-density-seeded cells in growth media; a minimal amount of expression was detected in high density culture upon osteogenic induction by ODM (Low: low density seeding culture; High: high density seeding culture). D) Quantitative analysis of scanned images of western blot. Graphs demonstrated a higher expression of CTGF and RhoA in low-density-seeded cells (ODM: osteogenic differentiation media; Growth: growth media).

To further determine which specific growth factors were responsible for the observed paracrine effect, we performed microarray assays on cells that were seeded at two distinct densities (low and high). Twenty-four hours after seeding, RNA was isolated from low-density-seeded larger cells and high-density-seeded smaller cells. Based upon the data from microarray analysis, the expression of connective tissue growth factor (CTGF) was shown to be 17-fold higher in low-density-seeded, larger cells (**Figure S1** of microarray summary). To validate the results from microarray, quantitative real-time PCR was performed on a separate isolate of ASCs seeded at different densities. Data showed significantly higher expression of CTGF in large cells as compared to small cells (*p≤0.05) (**Figure 2A**).

In order to more precisely determine the effect of cell size and area, we applied a micropatterning technique to quantitatively control the size of the cells. As described previously, the technique allowed us to compare large cells (50–100 um) to small cells (10–20 um) and evaluate CTGF expression. ASCs were allowed to spread into different, defined sizes of fibronectin coated islands (10 um∼100 um in diameter) in growth media. To detect the CTGF expression on larger and small cells, immunofluorescence staining was performed and intense staining of CTGF was shown on ASCs that were larger in size as compared to the small cells (**Figure 2B** **).** In addition, we detected up-regulation of RhoA (green) in low-density-seeded large cells, which provide an evidence of tight connection between small GTPase Rho A and CTGF. Interestingly, rhodamine phalloidin (red), which stained F-actin in the cells, showed the distinguished ruffled actin filament pattern in high-density-seeded small ASCs (**Figure 2B**). These data indicated that cell size manipulated by the seeding density resulted in an induction of CTGF as well as changes in actin cytoskeletal dynamics *in vitro*.

To follow up with the immunofluorescence staining, a semi-quantitative Western blot was performed on low-density-seeded and high-density-seeded cells in growth medium and in the presence of ODM. Data showed abundant CTGF protein expression in low-density-seeded ASCs as compared to high-density-seeded cells in both culture conditions (**Figure 2C**). Similarly, RhoA, an important mediator of mechanical force and cytoskeletal tension, was highly expressed in the low-density-seeded larger cells. Although ODM appeared to induce a trace amount of RhoA in high-density-seeded cells, the difference in RhoA expression between low- and high- density seeded cells remained substantial in the presence of ODM (**Figure 2C**). The image of the Western blot was analyzed semi-quantitatively with Image J software (**Figure 2D**). These data suggested that the cell size and its associated mechanical environment induced up-regulation of CTGF at both gene and protein levels, perhaps modulated by actin cytoskeletal tension through the RhoA pathway.

### Deficiency of CTGF expression affects osteogenic differentiation in ASC cells

**Figure 3:**
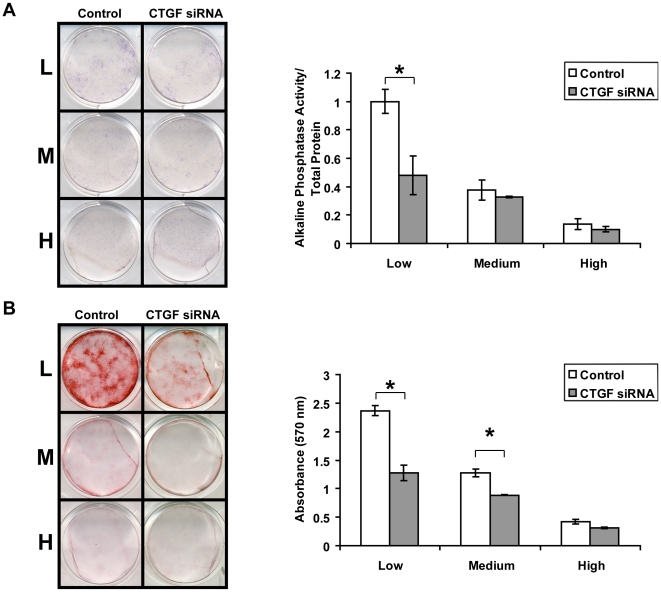
Changes in ASC osteogenic differentiation with CTGF knock-down. A) Alkaline phosphatase staining and quantification. Low-density-seeded ASCs transfected with CTGF-siRNA demonstrated decreased alkaline phosphatase activity compared to control (control siRNA-transfected ASCs) low-density-seeded cells (n = 3, *p≤0.05). B) Alizarin Red staining and quantification. CTGF inhibition with siCTGF carried longer term consequences in osteogenic differentiation. Alizarin red staining showed impaired late-stage osteogenesis in CTGF-deficient cells. Significant differences were observed at both low- and medium-density-seeded ASCs after CTGF knockdown (n = 3, *p≤0.05).

Given that up-regulation of CTGF in large cells was associated with osteogenic commitment in ASCs, we sought to assess the effect of CTGF deficiency in cytoskeletal tension-associated osteogenesis [27]. Utilizing a siRNA transfection in reduced-serum medium (Invitrogen), CTGF gene and protein expression was successfully knocked-down with approximately 80% efficiency (*p≤0.05) (**Figure S2**), as demonstrated by quantitative real-time PCR and Western blot, respectively. These ASCs were then differentiated in ODM at different seeding density. After one week, alkaline phosphatase staining and quantification of enzymatic activity was significantly decreased, particularly in large, low-density-seeded cells which expressed higher amounts of CTGF (**Figure 3A**). After two weeks of osteogenic differentiation, a significant decrease in mineralization was also observed by Alizarin Red staining and quantification in CTGF siRNA transfected cells as compared to the control transfected cells, particularly in large cells (**Figure 3B**). The differences were significant in low-density- and medium-density-seeded cells where CTGF was highly expressed upon seeding (*p≤0.05) (**Figure 3B**). Thus, knock-down of CTGF, a multifunctional matricellular protein, led to an insufficient signal transduction for mineralized ECM formation. As a result, these cells exhibited impaired osteogenesis.

### Alteration of Osteogenesis and Adipogenesis by Disruption of Cytoskeletal Tension with Rho/Rock Inhibitors

**Figure 4:**
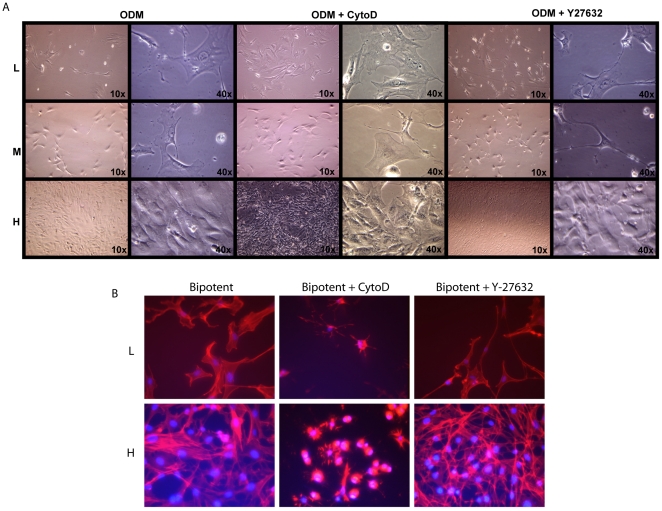
Changes in ASC morphology and differentiation with Rock/RhoA inhibitors. A) Microscopic images of cells plated at a low (L), medium (M) or high density (H) with and without addition of cytochalasin D or Y-27632. Cytochalasin D disrupted cell tension (resulting in flat cells) and significantly changed the morphologies of ASCs at all seeding densities. Y-27632 inhibited the downstream target Rock and did not lead to changes in cell morphology in either low-, medium- or high- density conditions (40×). B) Changes in ASC morphology with RhoA/Rock inhibitors. Actin filaments were stained with phalloidin conjugated to rhodamine (red) and counterstained with DAPI for cell nuclei (blue). Cells treated with cytochalasin D were small with disrupted actin structures in both low and high seeding densities, while treatment with Y-27632 had only a modest effect on ASC morphology.

Cell size as a result of plating density may affect mesenchymal cell differentiation, which was observed to be a RhoA mediated actin cytoskeletal tension associated function. Hence, we applied cytochalasin D, chemical inhibitor of actin polymerization to block the cytoskeletal tension; and Y-27632, an inhibitor of Rock, the downstream target of RhoA. These two reagents were used to block cytoskeletal tension at different stages and to examine their possible roles in osteogenic and adipogenic differentiation. As shown in **Figure 4A**, the morphologies of ASCs were dramatically changed upon the treatments of cytochalasin D and Y-27632. For example, cytochalasin D (0.5 ug/ml) disrupted cytoskeletal filaments, resulting in small cells in all seeding densities; however, Y-27632 (10 uM) blocked Rock, the downstream of RhoA, only showed modest effect on ASC morphology. The profound effect of cytochalasin D on cell size and morphology indicated that the cytoskeletal tension was also altered by blocking RhoA pathway.

Subsequently, we observed changes in ASC morphology with RhoA/Rock inhibitors. Actin filaments were stained with phalloidin conjugated to rhodamine (red) and counterstained with DAPI for cell nuclei (blue). Cells treated with cytochalasin D were small with disrupted actin structures in both low and high seeding densities, while treatment with Y-27632 had only a modest effect on ASC morphology (**Figure 4B**).

**Figure 5:**
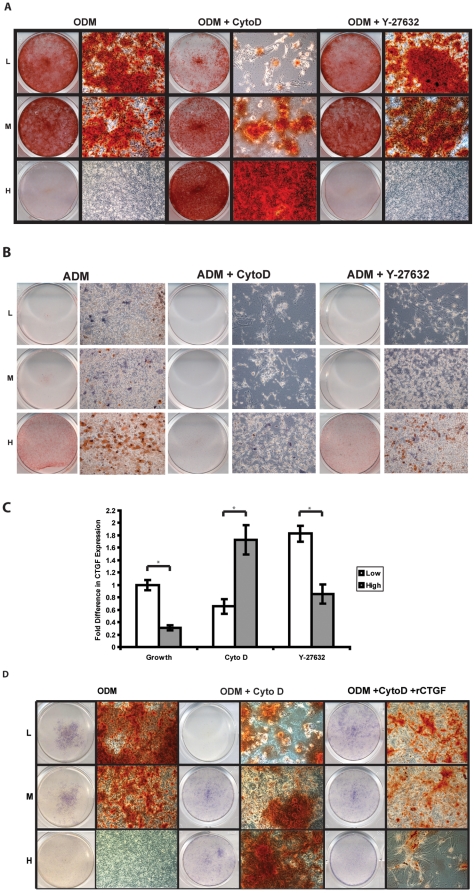
Characterization of cytoskeletal tension and regulation of CTGF. A) ASC osteogenic differentiation with and without cytochalasin D or Y-27632. At two weeks of osteogenic differentiation, robust matrix mineralization was observed in low-density-seeded cells as compared to high-density-seeded cells. With addition of cytochalasin D, the trend of osteogenic differentiation was reversed showing significant mineralization in high-density-seeded cells as compared to the control model. However, the addition of Y-27632 did not change the staining pattern. B) ASC adipogenesis with and without cytochalasin D or Y-27632. Addition of cytochalasin D in adipogenic medium abrogated the adipogenesis demonstrated by diminished Oil Red O staining. In contrast, Y-27632 did not affect the adipogenesis. C) Real-time PCR of CTGF expression pattern with and without cytochalasin D or Y-27632. Treatment of cytochalasin D diminished the CTGF expression in large cells in which CTGF was induced while spreading. Conversely, the expression of CTGF in small cells was up regulated showing completely different pattern of expression as compared to the initial seeding stage. In contrast, Y-27632 did not affect the CTGF expression pattern in different density seeded cells. D) Recombinant CTGF on osteogenesis. Alkaline phosphatase and Alizarin Red staining demonstrated that rCTGF (100 ng/ml) partially rescued the osteogenic capacity on the cells with Cytochalasin D treatment. The osteogenic capability was retained in low- and medium- density seeded cells determined by early alkaline phosphatase and late mineralization.

To explore the influence on differentiation, osteogenesis was induced by treating different density-seeded cells with ODM in the presence of cytochalasin D and Y27632. In the control group, as previously observed, low-density-seeded cells underwent robust osteogenesis as indicated by intense Alizarin Red staining whereas the high-density-seeded cells showed no mineralization. Interestingly, cytochalasin D treatment (0.5 ug/ml) in ODM completely altered the osteogenic effect caused by cell size. However, inhibition of downstream Rock by Y-27632 (10 uM) in ODM did not affect this cell size related osteogenesis (**Figure 5A**). Meanwhile, adipogenic differentiation was also assayed by using adipogenic medium (ADM) with Rock/Rho inhibitors on different density-seeded cells. We found that adipogenesis was completely blocked by cytochalasin D (0.5 ug/ml) but not Y-27632 (10 uM) (**Figure 5B**). Thus, these data suggested that cell size related osteogenesis and adipogenesis are directly modulated by the RhoA mediated action cytoskeletal tension.

Given that cytochalasin D robustly affected cell morphology and differentiation (low density-seeded cells showed diminished mineralization; high density- seeded cells showed increased mineralization), we next explored how CTGF was involved in this cytoskeletal tension associated function (**Figure 5C**). Quantitative real-time PCR demonstrated that the up-regulation of CTGF in low density-seeded large cells was diminished by treatment of cytochalasin D. This correlated with more mineralization observed in large cells (**Figure 5A**). Conversely, cytochalasin D increased mineralization in high density-seeded small cells and there was a dramatic increase of CTGF in cytochalasin D treated cells. On the other hand, the pattern of CTGF expression remained unchanged with treatment of Rock inhibitor Y-27632 (high CTGF expression in large cells and low CTGF expression in small cells) (**Figure 5C**). Thus, we concluded that the expression of CTGF was altered by blocking RhoA mediated actin cytoskeletal tension and the expression of CTGF contributes to osteogenesis in ASCs.

Next, in order to determine the specific role of CTGF in regulating the actin cytoskeletal tension associated osteogenesis, we supplemented the recombinant CTGF (100 ng/ml) in ODM to different density-seeded cells that are treated with and without cytochalasin D. Data showed that recombinant CTGF could partially rescue the osteogenesis modulated by the cytoskeletal tension (**Figure 5D**).

## Discussion

Strategies for directing mesenchymal stromal cell (MSC) differentiation are not yet well defined. The emergent physical microenvironment or niche where MSCs reside largely contributes to the regulation of lineage commitment [28]–[30]. Primarily isolated ASCs consist of osteo-, chondro- and adipogenic progenitors which will commit to their specific lineages under particular circumstances [31]–[33]. Recently, promising studies have explored the broad applications in using ASCs as a cell source for tissue engineering of cartilage and bone [34], [35]. Thus, the fundamental biology of these cells remains a current focus for translational research.

The transfer of mechanical strain results in activation of diverse signaling cascades, culminating in the reprogramming of certain gene expression and the production of growth factors and cytokines [27]. Mcbeath *et al*. proposed that soluble factors secreted from the MSCs were regulated by cell size associated cytoskeletal tension and maintained tissue homeostasis [14]. However, little is known about which growth factors contribute to the mechano-transactivation. Herein, utilizing *in vitro* manipulation of cell size by seeding at different densities, we showed dynamic differences in ASC morphology and actin cytoskeletal tension demonstrated by RhoA kinase and F-actin expression patterns. These differences were observed even though ASC growth was largely unaffected (**Figure S3**). Subsequently, differentiation potentials (osteogenesis and adipogenesis) were influenced by cell size and their associated microenvironment. Furthermore, we identified CTGF, a mechano-sensitive gene, which provides unique binding domains for growth factors (i.e. TGF-beta) and integrins, is involved in this cytoskeletal tension-associated ASC differentiation.

CTGF gene expression is known to be induced through mechanical stimulation and in particular through mechanical stretch. For example, *in vitro* uniaxial tension increases the expression of CTGF in chondrocytes, fibroblasts, and osteocytes [36]–[38]. *In vivo*, CTGF is upregulated in stretched tendon and in distraction osteogenesis of various cell types [39], [40]. Additionally, mechanical stretch stimulates osteogenic differentiation: stretched BMSCs have greater osteogenic gene expression, increased alkaline phosphatase activity, and enhanced mineralization [41]–[44]. Studies have shown that diminishing CTGF expression affects primarily the skeletal development as a result of impaired skeletal proliferation and ECM production. Consistent with these *in vivo* findings, we have demonstrated that CTGF deficiency *in vitro* is sufficient to influence osteogenesis. Since CTGF does not work independently, other growth factors, integrin family proteins and possible regulatory mechanisms linking this signaling cascade remain to be investigated.

Most mechanical sensors are integrin family proteins linking ECM proteins to intracellular signaling. Modulations of these ECM proteins are coupled with intergrin-linked kinases such as small GTPases [15]. The small GTPases of the Rho family are central in mechano-transduction mediating the formation of focal complexes, transducing signals intracellularly, and subsequently inducing changes in gene expression, cellular shape and morphology [15]. In our study, blocking the arrangement of actin cytoskeletal tension by cytochalasin D led to the reorganization of cytoskeletal proteins that predominately influence cell tension and cell size in ASCs. The rearrangement of actin cytoskeletal tension subsequently influenced the lineage differentiation in these cells. Although inhibiting ROCK, the down stream target of RhoA demonstrated a modest change in ASC morphology, the differentiation capacity was not significantly affected. Therefore, we concluded that RhoA mediated actin cytoskeletal tension largely contributes to the regulation of mesenchymal cell diffrentiation.

The molecular connection between the RhoA mediated actin cytoskeletal tension and CTGF expression was revealed by examining the expression of CTGF in cells after the treatment of cytochalasin D. CTGF was significantly suppressed by the disruption of RhoA mediated cytoskeletal tension. As a result of effect, these cells were directed into alternative lineages. Given the fact that recombinant CTGF partially restored this osteogenic capability, we propose that multiple signaling cascades are involved in this RhoA mediated cytoskeletal tension associated osteogenesis and influence the cell fate decision in such microenvironment [45], [46]. Although we have demonstrated a novel function of CTGF in our cell system, diverse signaling into the molecular and cellular interaction between CTGF and Rho/Rock pathway remains to be further elucidated.

Through our microarray analysis, multiple molecular signaling pathways were identified to be potentially involved and complex interdependent signaling networks are likely coordinated into this cytoskeletal tension-associated regulation. In addition to up-regulation of CTGF, our data showed that calponin-2, an actin-binding protein implicated in cytoskeletal reorganization was expressed five times higher in large cells [47], [48]. Other studies have demonstrated that calponin-2 expression was increased in bone morphogenesis with retinoic acid-induced osteoblastic differentiation [47]. Furthermore, colony stimulating factor-1 (CSF-1), tropomyosin-2 (TM-2) and SWI/SNF were all increased over three fold in large cells. These genes are well described in either bone remodeling or actin reorganization [49]–[51]. In contrast, chemokine (C-X-C motif) ligand 12/SDF-1was highly expressed in small cells seeded at a high density. This gene was proved to be associated with hypoxia gradient response and contributes to maintaining cells in undifferentiated stage [52], [53]. These data highlighted the formation of cytoskeletal tension with multiple interdependent signaling cascades.

Understanding *in vitro* models of micromechanical regulation will advance the knowledge of how ASCs respond to various cellular signaling. Fully comprehending these mechanisms can bridge the chasm between developmental biology and tissue regeneration.

## Supporting Information

Figure S1 Results of microarray analysis of up-regulated and down-regulated genes in low-density-seeded cells. Table lists the genes that show substantial expression differences in low-density-seeded cells as compared to high-density-seeded cells. Positive fold changes indicate up-regulation in low-density-seeded, large cells. Negative values indicate down-regulation of the genes in the low-density-seeded cells.
(0.03 MB DOC)

Figure S2 Efficiency of the CTGF knock-down by siRNA transfection. A) Quantitative real-time PCR analysis after 48 hours of transfection. Approximately 80% of decrease in CTGF gene expression was shown (*p<0.05). B) Western blot analysis of CTGF protein expression. CTGF protein expression was significantly decreased after CTGF SiRNA transfection. Minimal amount of CTGF expression was detected after 48 hours of transfection.
(4.75 MB TIF)

Figure S3 MTT assay for cell proliferation analysis (n = 3 wells per condition). At day 7, cell proliferation had increased in all density conditions with similar growth overtime.
(7.23 MB TIF)
