# Supplementary material for: Connective Tissue Growth Factor in Regulation of RhoA Mediated Cytoskeletal Tension Associated Osteogenesis of Mouse Adipose-Derived Stromal Cells
Source: PLoS One. 2010 Jun 23;5(6):e11279. doi: 10.1371/journal.pone.0011279 (PMC2890586; doi:10.1371/journal.pone.0011279)
Supplement: Figure S1 — Results of microarray analysis of up-regulated and down-regulated genes in low-density-seeded cells. Table lists the genes that show substantial expression differences in low-density-seeded cells as compared to high-density-seeded cells. Positive fold changes indicate up-regulation in low-density-seeded, large cells. Negative values indicate down-regulation of the genes in the low-density-seeded cells. (0.03 MB DOC) [file pone.0011279.s001.doc]

**Supplemental Figure 1 (Table 1):**

| **Gene** | **Fold Change** |
| --- | --- |
| **Connective tissue growth factor** | **+17.38** |
| **Calponin 2** | **+5.29** |
| **Colony stimulating factor-1** | **+3.89** |
| **Tropomyosin 2** | **+3.49** |
| **SWI/SNF related, matrix-associated, actin dependent regulator of chromatin, subfamily b, member 1** | **+3.58** |
| **Chemokine (C-X-C motif) ligand 12** | **-9.56** |
| **Secreted frizzled-related protein 2** | **-7.44** |
| **Insulin-like growth factor binding protein 4** | **-4.48** |
|  |  |
