# Supplementary figures and images for: Connective Tissue Growth Factor in Regulation of RhoA Mediated Cytoskeletal Tension Associated Osteogenesis of Mouse Adipose-Derived Stromal Cells
Source: PLoS One. 2010 Jun 23;5(6):e11279. doi: 10.1371/journal.pone.0011279 (PMC2890586; doi:10.1371/journal.pone.0011279)

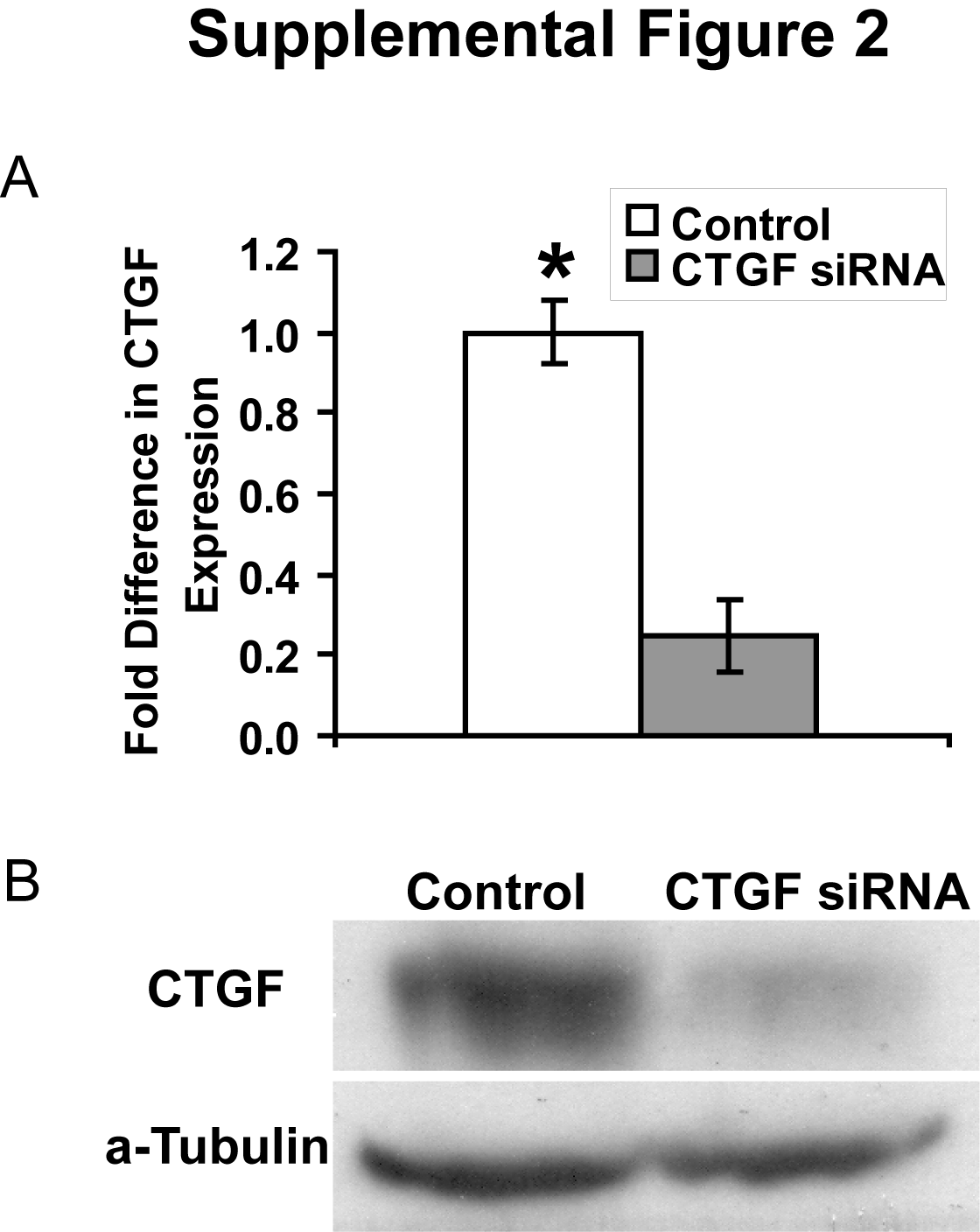

Supplement: Figure S2 — Efficiency of the CTGF knock-down by siRNA transfection. A) Quantitative real-time PCR analysis after 48 hours of transfection. Approximately 80% of decrease in CTGF gene expression was shown (*p<0.05). B) Western blot analysis of CTGF protein expression. CTGF protein expression was significantly decreased after CTGF SiRNA transfection. Minimal amount of CTGF expression was detected after 48 hours of transfection. (4.75 MB TIF) [file pone.0011279.s002.tif]

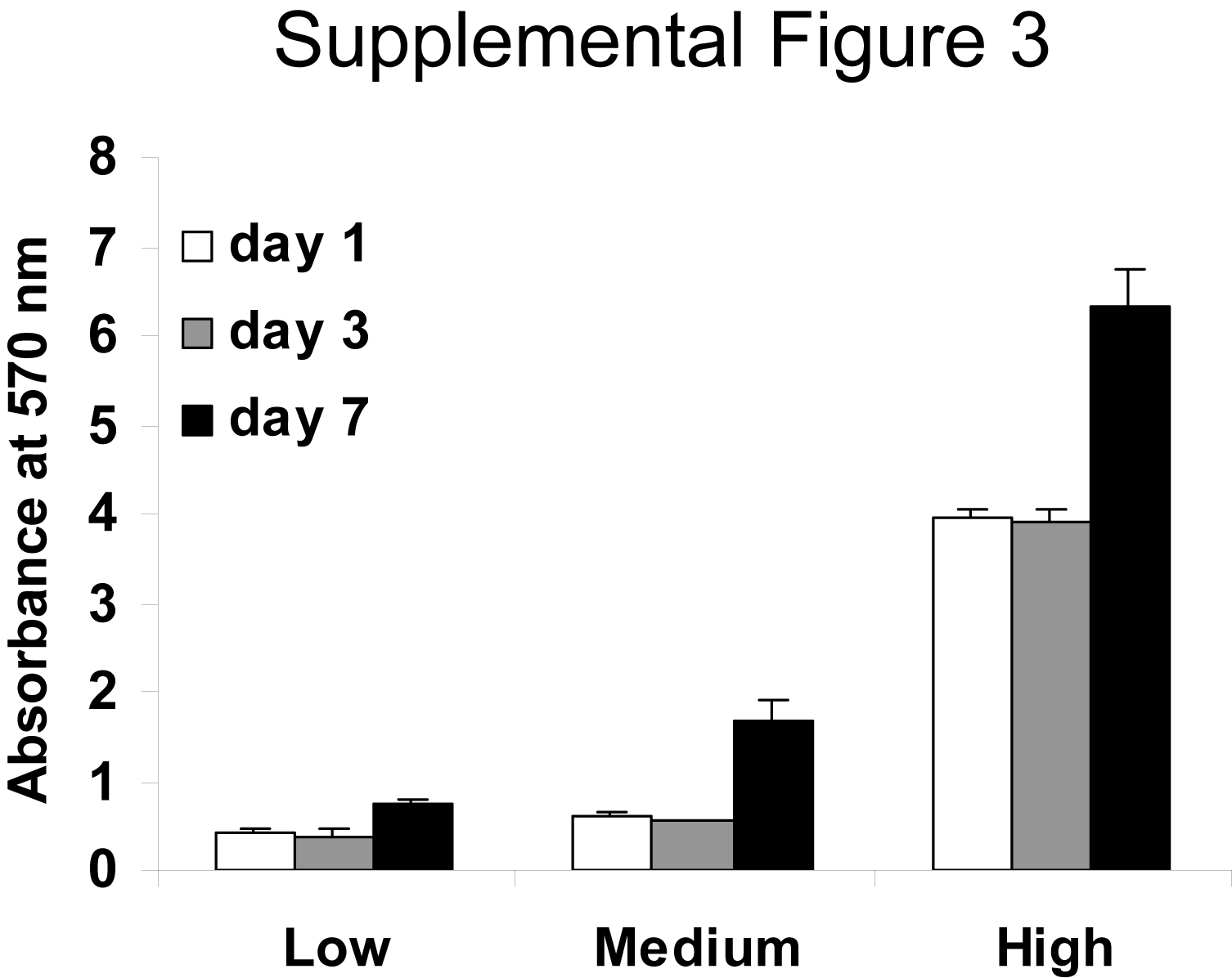

Supplement: Figure S3 — MTT assay for cell proliferation analysis (n = 3 wells per condition). At day 7, cell proliferation had increased in all density conditions with similar growth overtime. (7.23 MB TIF) [file pone.0011279.s003.tif]
